# A novel optical tracer for VMAT2 applied to live cell measurements of vesicle maturation in cultured human β-cells

**DOI:** 10.1038/s41598-019-41891-x

**Published:** 2019-04-01

**Authors:** Stevan Pecic, Nenad Milosavic, Gina Rayat, Antonella Maffei, Paul E. Harris

**Affiliations:** 1 Department of Chemistry and Biochemistry, California State University, Fullerton, California, USA; 2 Division of Experimental Therapeutics, Department of Medicine, Columbia University Medical Centre, New York, New York, USA; 3 Alberta Diabetes Institute, Ray Rajotte Surgical-Medical Research Institute, Department of Surgery, University of Alberta, Edmonton, AB T6G 2E1 Canada; 4 Division of Endocrinology, Department of Medicine and Naomi Berrie Diabetes Center, Columbia University Medical Centre, New York, New York, USA

## Abstract

The islet β-cells integrate external signals to modulate insulin secretion to better regulate blood glucose levels during periods of changing metabolic demand. The vesicular monoamine transporter type 2 (VMAT2), an important regulator of CNS neurotransmission, has an analogous role in the endocrine pancreas as a key control point of insulin secretion, with additional roles in regulating β-cell differentiation and proliferation. Here we report on the synthesis and biological characterisation of a fluorescent ligand for VMAT2 suitable for live cell imaging. Staining for VMAT2 and dopamine in live β-cell cultures show colocalisation in specific vesicles and reveal a heterogeneous population with respect to cell size, shape, vesicle number, size, and contents. Staining for VMAT2 and zinc ion, as a surrogate for insulin, reveals a wide range of vesicle sizes. Immunohistochemistry shows larger β-cell vesicles enriched for proinsulin, whereas smaller vesicles predominantly contain the processed mature insulin. In β-cell cultures obtained from nondiabetic donors, incubation at non-stimulatory glucose concentrations promotes a shift in vesicle diameter towards the more mature insulin vesicles at the expense of the larger immature insulin secretory vesicle population. We anticipate that this probe will be a useful reagent to identify living β-cells within complex mixtures for further manipulation and characterisation.

## Introduction

The islet β-cells integrate external signals to modulate insulin secretion to fine tune blood glucose levels during periods of changing metabolic demand^1^. In addition to glucose, fatty acids and gut derived peptides, net insulin production is regulated by a number of other molecules, including a number of classical neurotransmitters including dopamine. We and others have shown that β-cell secreted dopamine (DA) mediates a glucose stimulated insulin secretion (GSIS) inhibitory circuit in human β-cells^2–4^. We demonstrated that islet β-cells co-secrete insulin and dopamine in response to glucose stimulation, both *in vivo* and *in vitro*, and that dopamine down regulates insulin secretion via dopamine type 2 like-receptors (D2R) expressed by β-cells. In this circuit, DA, synthesised de novo or imported by dopamine (reuptake) transporter (DAT), is stored in β-cell vesicles by the action of the vesicular monoamine transporter type 2 (VMAT2). These insulin granules also contain D2R. During GSIS, DA and insulin are released and D2R is delivered to the cell surface where it binds DA to down-regulate insulin secretion by an autocrine mechanism^5^. Thus, in the pancreas, VMAT2 is a central point for control of insulin secretion, a function analogous to its role in the CNS of regulating monoamine neurotransmission^6^.

VMAT2 is a tissue restricted marker of β-cells in the pancreas. Previously, VMAT2 quantification in living cells had only been performed at the population level *in situ* by positron emission tomography (PET)^7–9^, or by indirect methods relying on non-specific fluorescent substrates of vesicular monoamine transporters^10,11^. PET imaging of human β-cells *in situ* relies on [18 F] or [11 C] labelled dihydrotetrabenazine. Dihydrotetrabenazine ((+) DTBZ) is a VMAT2 ligand with a nanomolar affinity constant^12^. We set out to develop a (+) DTBZ based VMAT2 ligand with a fluorescent reporter suitable for live cell imaging and tested its utility in morphometric studies of β-cell vesicles.

## Results

### Physiochemical characterisation

**Figure 1:**
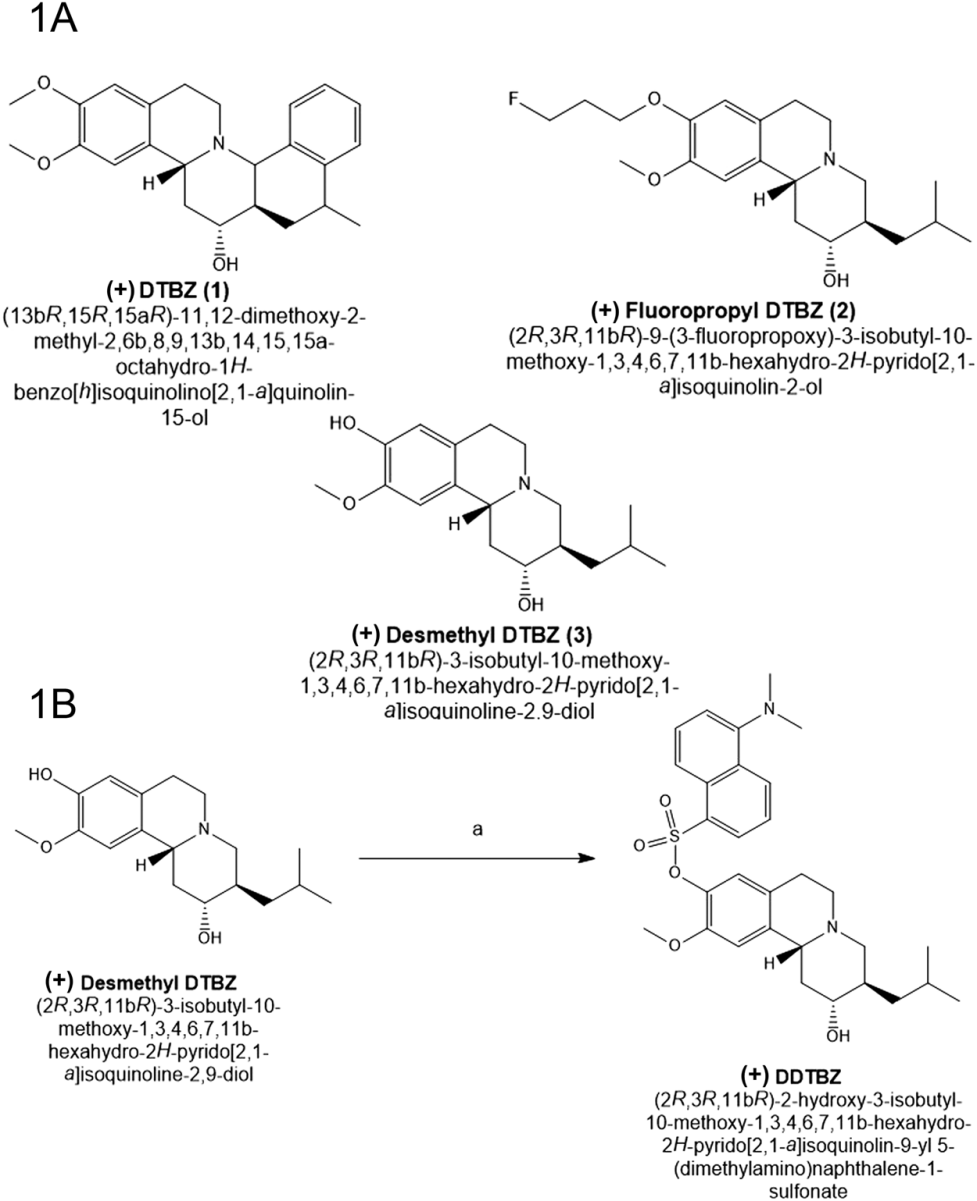
Structures and Synthesis of (+) DDTBZ. Panel 1A Dihydrotetrabenazine based structures. (1) Dihydrotetrabenazine (2-hydroxy-3-isobutyl-9-methoxy-10 -methoxy-1,2,3,4,6,7,- hexahydro-11bH-bezo[alpha]-quinolizine) ((+) DTBZ). (2) (+)-2-Hydroxy-3-isobutyl-9-(3-fluoropropoxy)-10-methoxy-1,2,3,4,6,7-hexahydro-11bH-benzo[a]quinolizine ((+) FPDTBZ). (3) 2H-Benzo[a]quinolizine-2,9-diol, 1,3,4,6,7,11b-hexahydro-10-methoxy-3-(2-methylpropyl)-, (2 R,3 R,11bR) ((+) Desmethyl DTBZ). Panel 1B Synthesis of 2-hydroxy-3-isobutyl-9-methoxy-1,3,4,6,7,11b-hexahydro-2H-pyrido[2,1-a]isoquinolin-8-yl 5-(dimethylamino)naphthalene-1-sulfonate ((+) DDTBZ) reagents and conditions: (a) 5-(dimethylamino)naphthalene-1-sulfonyl chloride (dansyl chloride), N,N-dimethylpyridin-4-amine (DMAP), dichloromethane (DCM), 0 °C, RT, room temperature.

The synthetic strategy for the probe was based on the structures of the specific VMAT2 inhibitor dihydrotetrabenazine ((+) DTBZ), the validated, subnanomolar Kd PET probe for VMAT2, 18F-fluoropropyl dihydrotetrabenazine (FPDTBZ), the radiosynthetic precursor of 18F-FPDTBZ, (+)-9-O-Desmethyl-α-Dihydrotetrabenazine (Fig. 1A,B).

**Figure 2:**
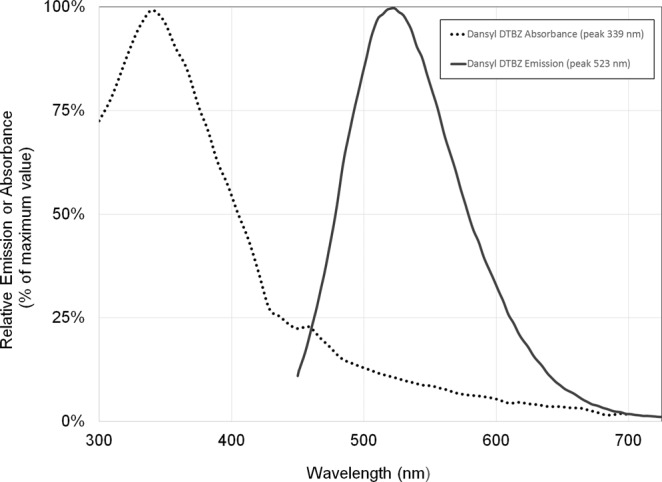
Excitation and emission spectra of (+) DDTBZ. Stock solutions of (+) DDTBZ in DMSO were diluted to 20 uM and their excitation-emission spectra recorded. Results are diluent (PBS, 1% DMSO) background subtracted.

The probe synthesised had a molecular weight of 538 g/mol by ESI-MS (m/z + 1 = 539). Characterisation of absorption and emission spectra of dansyl (+)DTBZ ((+) DDTBZ) revealed maxima at λ_ex_ = 339 nm and λ_em_ = 523 (Fig. 2). As expected, the excitation and emission maxima were similar to the parent fluorescent reporter dansyl chloride. Neither the radiosynthetic precursor nor DTBZ showed significant fluorescence at 523 nm at the concentrations tested (≤100 µM).

### (+) DDTBZ colocalises with and binds preferentially to VMAT2 positive cells

**Figure 3:**
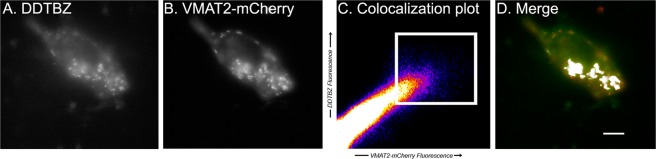
(+) DDTBZ binding colocalises with mCherry-VMAT2 and binds preferentially to VMAT2 transfected HEK 293 cells. Successive z focal planes of a HEK-DAT mCherry-VMAT2 cell stained with (+) DDTBZ (Panel A–H). (+) DDTBZ (30 µM) was added to cell cultures, cells were incubated and then washed and imaged (excitation at 385 nm, emission collected at 465–525 nm) (Panel A). The VMAT2-mCherry fusion protein was visualised at 645–720 nm (Panel B). Panel C is the colocalisation plot for the data collected in Panels A and B. - The z axis of Panel C is the heat map scoring of the number of pixels with equivalent intensities. The pixel intensities in each channel laying within the region of interest (white box) region of interest were significantly correlated (p = 0.86) and are shown in white in Panel D. the merged pseudocolour image of Panels A (green) and B (red) and region of interest data from Panel C (within white box). Scale bar represents 10 µm.

To demonstrate the specificity of (+) DDTBZ to VMAT2, various concentrations of (+) DDTBZ were added to live cultures of HEK 293 transfected with the VMAT2-mCherry fusion protein. Cells were then imaged for (+) DDTBZ fluorescence signal, followed by VMAT2-mCherry fluorescence at the indicated wavelength (Fig. 3).

**Figure 4:**
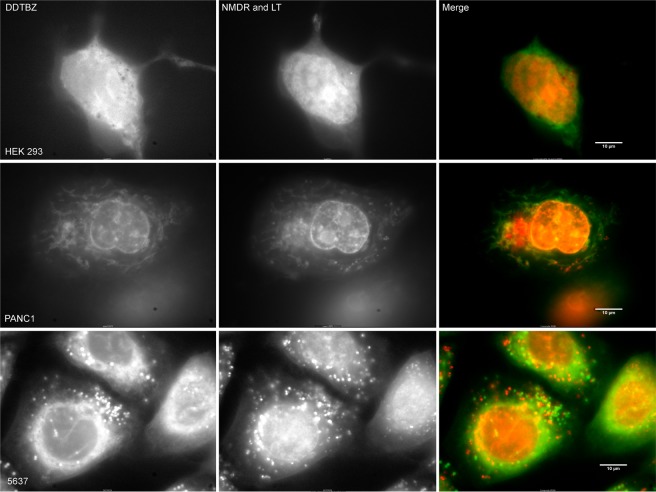
Vesicular binding of (+) DDTBZ distinguishes VMAT2 positive cells from the non-specific binding pattern in VMAT2 negative cells. HEK293, PANC1 and 5637 cells were stained with (+) DDTBZ, Nuclear Mask Deep Red (NMDR) and Lysotracker Red DND-99  (LT). The (+) DDTBZ signal was acquired with a 495BP30 filter and is shown in green in the pseudocolour merged images at right. NMDR and LT signals were imaged together in a 655 to 710 nm window and are shown in red in the pseudocolour merged images at right. Scale bar represents 10 microns.

We found (+) DDTBZ signal significantly overlapped with mCherry VMAT2 signal. Next, we extended the studies of (+) DDTBZ binding to three other non-beta cell lines to characterize the possible specific and non-specific binding of the probe. HEK 293 cells, the untransfected counterparts of HEK-DAT mCherry-VMAT2 cells studied above, PANC1 cells, derived from a human pancreatic carcinoma of ductal cell origin and 5637 cells, derived from a human uroepithelial carcinoma were seeded into coverglass chambers slides and allowed to attach and proliferate. HEK 293, PANC1 and 5637 cells were incubated with (+) DDTBZ, LysoTracker Red and Nuclear Mask Deep Red and the cells imaged at the indicated wavelengths (Fig. 4). The collection of DDTBZ fluorescent signal was performed under a higher gain setting relative to that used for imaging, LysoTracker Red and Nuclear Mask Deep Red signals, to allow visualization of possibly weak signals. We found that (+) DDTBZ fluorescence in HEK 293 cells was greatest in the nuclear region and perinuclear regions of the cells, but not found in vesicles. LysoTracker red staining of HEL 293 cells show few vesicles. Similarly (+) DDTBZ fluorescence in PANC-1 cells was associated with the nucleous and some uncharacterized cytoplasmic structures. LysoTracker red staining of PANC-1 cells show many more small vesicles than HEK 293, but (+) DDTBZ fluorescence was not associated with these vesicles. The 5637 cell line showed abundant vesicular staining with (+) DDTBZ, as well as perinuclear and nuclear staining. LysoTracker red staining of 5637 cells show many more small vesicles than PANC-1 cells. (+) DDTBZ vesicular fluorescence was associated with a partially overlapping set of Lysotracker red avid vesicles, but some vesicles stained only with (+) DDTBZ. Using the greyscale values from the cell images, the vesicular staining in 5637 cells was 2–5 times the cytoplasmic background depending on the location of the vesicles. Examination of the public gene expression databases revealed that HEK 293 cell do not express SLC18A2 transcripts (a.k.a VMAT2)^13^, while PANC-1 cells express SLC18A2 transcripts weakly (i.e. in the bottom 0.3% of the >40 k different genes probed)^14^. In a previous report, 5637 cells were used to deposit an extracellular matrix on cover slips suitable for human beta cell culture^15^. 5637 cells have also been used a source of secreted colony stimulating factors^16^, but were not recognized for expression of VMAT2. The vesicular staining by (+) DDTBZ correctly identified 5637 cells as expressing SLC18A2 transcripts^17^.

**Figure 5:**
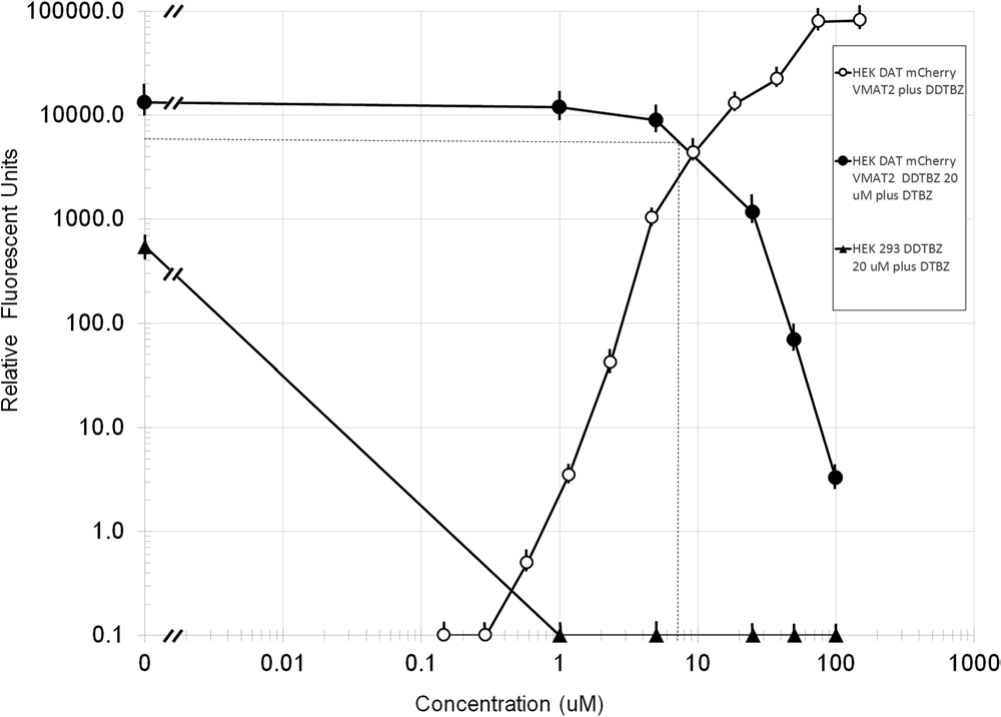
Specificity, Displaceability and Saturability of (+) DDTBZ binding. HEK-DAT-mCherryVMAT2 or untransfected HEK 293 cells were cultured in 96 well glass bottom black plastic plates. (+) DDTBZ (20 µM) was added to the cultures and the bound fluorescence quantified. Results are the averages of three independent experiments. The IC_50_ (DDTBZ by ((±) DTBZ ≈ 7 µM) was estimated graphically (dotted line). Error bars are the standard error of the mean.

To better quantify the displaceability and saturability of (+) DDTBZ binding to cellular VMAT2, we repeated the binding experiments in 96 well plates suitable for fluorescent imaging of HEK-DAT mCherry-VMAT2 and untransfected HEK 293 cells. Cell cultures were incubated in (+) DDTBZ with and without (±) DTBZ, washed and the bound fluorescence signal quantified (Fig. 5). Binding of (+) DDTBZ was saturable, and displaceable by increasing concentrations of (±) DTBZ in HEK -DAT, mCherry-VMAT2 transfected cells. Using this platform, the HEK-DAT mCherry VMAT2 cells bound >50 times more (+) DDTBZ relative to the control, untransfected HEK 293 cells.

### VMAT2 and dopamine colocalise in the vesicles of cultured β-cells

**Figure 6:**
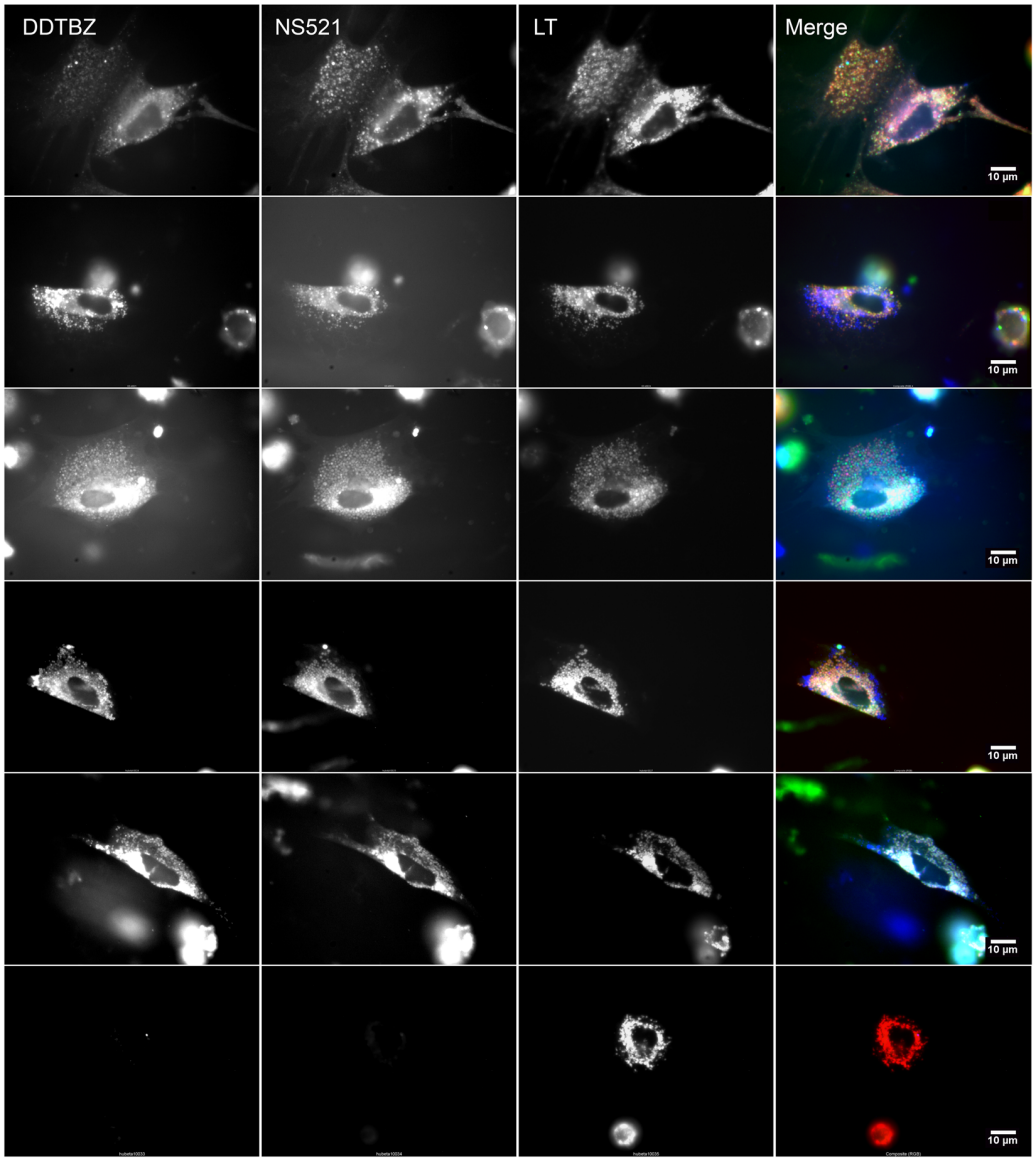
(+) DDTBZ, NeuroSensor 521 and LysoTracker® Red DND-99 staining of live human islet cell cultures identifies VMAT2 positive, monoamine positive, acidic vesicles in a morphologically diverse population of cells. Cell cultures were stained with the three probes and imaged in their corresponding channels. These are representative images from identical experiments performed on two different donors 991 and 025. The merged pseudocolour image (right panels) represents the three grey scale images, (+) DDTBZ (blue channel), NeuroSensor 521 (green channel) and Lysotracker Red DND-99 (red channel) translated to an RGB image. Scale bar represents 10 µm.

We previously demonstrated that β-cells are the primary storage site for dopamine in the pancreas^18^. Both insulin and VMAT2 immunoreactivity colocalise almost exclusively in the β-cells of human islets^19,20^, and human islets release both insulin and dopamine in response to glucose challenge^2^. We took advantage of the two-dimensional human β-cell culture system described by Phelps *et al*.^15^ to examine the expression of VMAT2 in dissociated human and porcine islet cell cultures. Since (+) DDTBZ excitation spectrum peaks at near UV wavelengths, we were able to use other fluorescent probes for imaging the same cultures. NeuroSensor 521 is a fluorescent turn-on sensor that allows for the selective recognition and sensing of norepinephrine and dopamine in live cells^21^. Since islet β-cells do not express Dopamine-Beta-Hydroxylase responsible for the transformation of dopamine into norepinephrine^22^ and analysis of human islet culture supernatants by HPLC coupled with electrochemical detection demonstrate dopamine but fail to show significant amounts of norepinephrine (unpublished observations), we concluded that NeuroSensor 521 would enable us to visualise the presence of vesicular dopamine (DA) in these cultures. β-cell cultures were also stained with LysoTracker® Red DND-99, a fluorescent probe specific for acidic organelles such as lysosomes as well as the insulin containing β-cell vesicles which are reported to have a resting pH around 5.9–6.2^23^. In Fig. 6 representative images of cells stained with (+) DDTBZ, NeuroSensor 521 and LysoTracker Red DND-99 are presented.

Merged pseudocolour images of (+) DDTBZ, NeuroSensor 521 and LysoTracker® Red DND-99 stained cultures revealed a morphologically heterogeneous population of cells with respect to cell shape, degree of probe binding and vesicle size. (+) DDTBZ, NeuroSensor 521, Lysotracker red binding represented largely overlapping sets, but many vesicles showed strong binding of only one or two of the fluorescent probes. Not all cells bound (+) DDTBZ and NeuroSensor 521 and are likely to represent the non-β-cell constituents of the islet cultures (see Supplemental Fig. S1).

Murine islets have been reported to express lower amounts of VMAT2 relative to its’ expression in human islets^24^. We examined the binding pattern of (+) DTBBZ in cultures of βTC-6, an insulin secreting mouse cell line derived from transgenic B6D2 mice harboring the SV40 T-antigen driven by the upstream enhancer-promoter region of the rat insulin II gene^25^. We found that (+) DDTBZ staining (Supplemental Fig. S2) was heterogenous as we could identify cells clusters devoid of (+) DDTBZ positive cells (Supplemental Fig. S2, upper panel), cell clusters containing both (+) DDTBZ positive and negative cells (Supplemental Fig. S2, lower panel) and clusters composed of almost entirely of (+) DDTBZ positive cells. Within the group of βTC-6 cells and cell clusters staining positively with (+) DDTBZ, the number of (+) DDTBZ positive vesicles per cell (mean = 2.2 versus 208) as well as the vesicle diameters (mean = 512 nm versus 699 nm) were significantly (p < 0.05, two tailed test) smaller than vesicles imaged in (+) DDTBZ positive human islet cells.

To demonstrate that beta cell vesicular binding of (+) DDTBZ was displacable, islet cell cultures treated with 30 µM (+) DDTBZ and an 8 fold molar excess of racemic (±) DTBZ (corresponding to a 4 fold molar excess of (+) DTBZ). Vesicles in these cultures showed an average 50% reduction in average vesicular fluorescent intensity (see Supplemental Fig. S3).Next, we examined cultures of dispersed porcine islet cells treated with (+) DDTBZ, NeuroSensor 521 and LysoTracker® Red DND-99 (see Supplemental Fig. S4). Similar results to human islets cultures were obtained.

### VMAT2 and Zinc colocalise in the vesicles of cultured β-cells

The role of zinc in the formation of mature insulin secretory vesicles has been reviewed by Li^26^. Briefly, translated preproinsulin in the lumen of the rough ER is cleaved to form proinsulin. Proinsulin is transported to the trans-Golgi network for packaging into secretory granules where it is converted to insulin by selective cleavage of the C-peptide. Insulin is initially found in its monomeric form, but as it accumulates, a dimeric conformation is favored. Intracellular Zn^+2^ is transported via ZnT8 into the vesicles where it facilitates the formation of a 2-Zn^+2^-trimer of dimers (i.e. hexameric insulin) in a process reported to be stabilised by the presence of dopamine^27^. On this basis, we have used intravesicular Zn^+2^ combined with the presence of VMAT2 as a surrogate marker for insulin containing vesicles.

**Figure 7:**
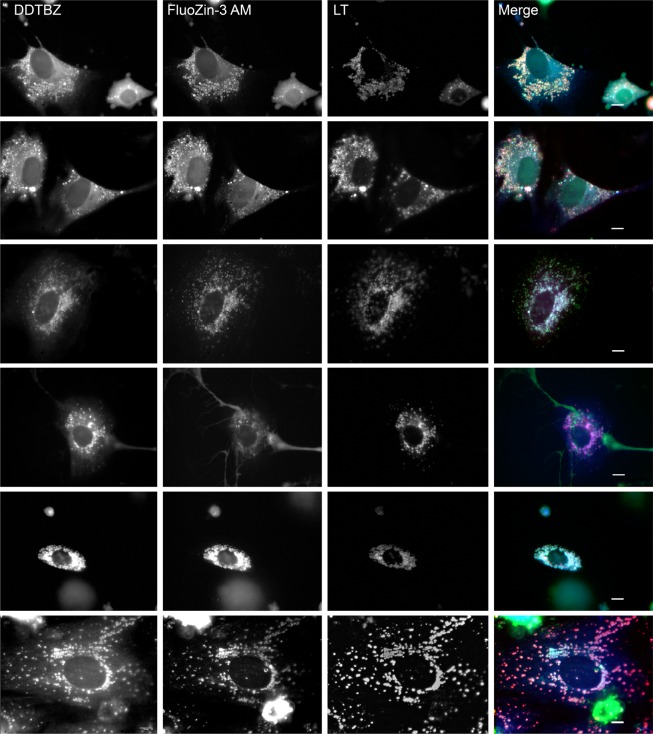
(+) DDTBZ, FluoZin™-3 AM zinc probe and Lysotracker staining identify a morphologically heterogeneous population of cells with VMAT2 positive, Zinc positive acidic vesicles in live human islet cultures. A merged pseudocolour image (right panels) represents the three grey scale images, (+) DDTBZ (blue channel), FluoZin-3 AM (green channel) and Lystotracker Red DND-99 (red channel) translated to an RGB image These are representative images from identical experiments performed on three donors (141, 212 and 556). Scale bar represents 10 µm.

Fluorescent zinc probes have been used to image intracellular Zn^+2^ in β-cells^28,29^ but not at the resolution levels used in current experiments. We selected FluoZin-3, AM, an on-off selective fluorescent Zn^+2^ probe^30^. The probe is prepared as an acetoxymethy ester which enhances cell permeability, yet de-esterifies intracellularly as an aid to maintenance of the signal. Merged pseudocolour images of (+) DDTBZ, FluoZin-3, AM and LysoTracker® Red DND-99 stained cultures (Fig. 7) revealed a morphologically heterogeneous population of cells with respect to cell shape, degree of probe binding and vesicle size. (+) DDTBZ, FluoZin-3, and LysoTracker Red triple positive vesicles could be identified in a majority of cells. In addition, we could identify at least three subsets of triple positive cells; 1) Cells containing mostly larger vesicles (diameters 600–2000 nm) (Fig. 7, row 1), 2) Cells with mixed large and small vesicles (Fig. 7, row 2), and 3) cells containing mostly smaller vesicles (diameters ≤480 nm) (Fig. 7, row 3). Some cells showed (+) DDTBZ positive vesicles, yet only a few vesicles binding FluoZin-3 and a weak cytoplasmic signal in the green channel (Fig. 7, row 4).

Lastly, common features of the β-cell vesicles visualised by (+) DDTBZ were the occasional apparent entrainment of vesicles within the β-cell cytoplasm (e.g. Fig. 7, row 1), a wide range of vesicle diameters, and perinuclear regions relatively void of vesicles staining with (+) DDTBZ (e.g. Fig. 7, row 1 and 2) compared to adjacent areas. We speculated that the larger vesicles might be the newly formed vesicles enriched for proinsulin polypeptide and the smaller vesicles enriched with the mature processed form insulin. Human β-cells cultures were fixed and processed for immunohistochemistry with anti-VMAT2 antibodies and anti-proinsulin or anti-insulin antibodies. Confirming our previous findings using formaldehyde fixed paraffin embedded whole pancreas tissue^19^, we found that VMAT2 and insulin immunoreactivity colocalised in the cytoplasm of fixed β-cells (see Supplemental Fig. S5). Additionally we found that larger VMAT2 positive vesicles were indeed enriched for proinsulin polypeptide (see Supplemental Fig. S6).

To better understand the relationship between (+) DDTBZ binding and the morphology of human β-cells in this culture system, we stained the β-cells with (+) DDTBZ and substituted FluoZin-3 AM with BODIPY™ FL C5-Ceramide complexed to BSA (see Supplemental Fig. S6). BODIPY™ FL C5-Ceramide is a well characterised stain for the trans-Golgi network^31^. We observed that the cytoplasmic voids of (+) DDTBZ positive vesicles represented areas containing the trans-Golgi network as revealed by the binding of BODIPY™ FL C5-Ceramide. Apparent nascent secretory vesicles, as judged by their intimate association with the Golgi membranes, were visible. Of these nascent vesicles, only a minority retained (+) DDTBZ or LysoTracker Red. (see Supplemental Fig. S6).

### Incubation of cultured β-cells in low glucose is accompanied by a reduction in average insulin vesicle size

**Figure 8:**
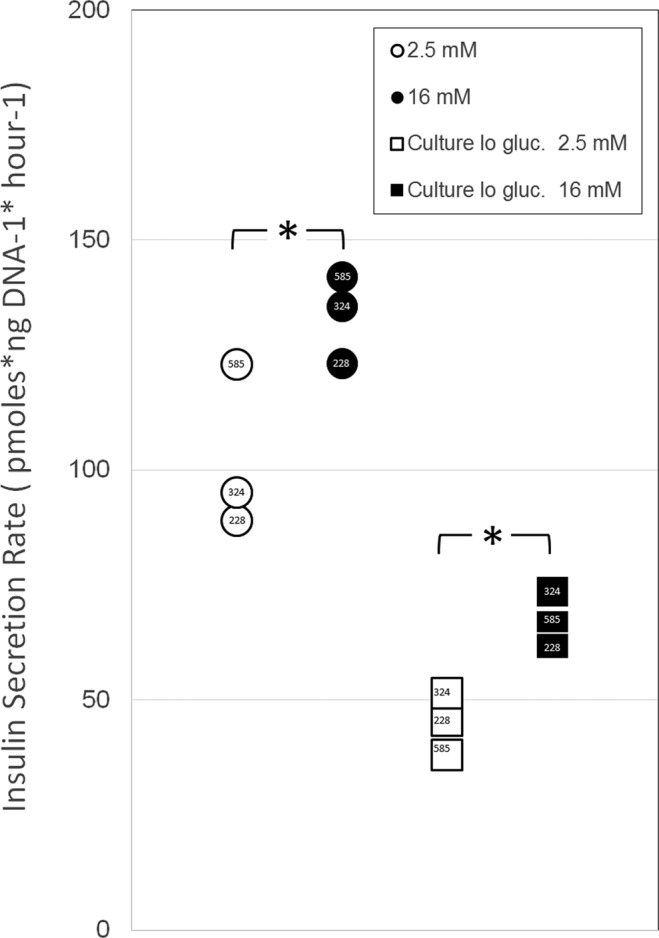
Insulin secretion characteristics of β-cell cultures. Panel A β-cell cultures were cultured in regular media containing 12 mM glucose for 5 days (Hi Gluc), then assayed for glucose stimulated insulin secretion (GSIS). Panel B β-cell cultures were cultured in regular media containing 12 mM glucose for 5 days, transferred to media supplemented to 4 mM glucose (Lo Gluc), then assayed for GSIS. The insulin content of supernatants was measured by ELISA and normalized to the DNA content of the well. Asterisks show statistical significance (p < 0.05, paired t test) of the difference between basal and stimulated insulin secretion. Donor RRID appears within each symbol.

*In vitro*, islets, β-cells, and β-cell lines are often cultured under growth/survival permissive moderate glucose concentrations (10–12 mM Glucose). For measurements of insulin secretion from these cultures, however, the normal culture media is often exchanged with a lower glucose concentration basal medium that does not trigger insulin secretion and the cultures are allowed to “rest” for various intervals, nominally to allow the accumulation of fully processed mature insulin secretory vesicles. Once stimulated, the amplitudes of secretory responses are greater relative to cultures that have not been preincubated in low glucose basal medium. We tested the insulin secretion characteristics of the two-dimensional dispersed human β-cell culture system. The β-cell cultures were incubated normally in 12 mM glucose culture medium for five days and then tested for glucose stimulated insulin secretion (GSIS), or cultured for an additional two in culture medium containing 4 mM Glucose. Cultured β-cells were washed and then rested for 60 minutes at 37 C in a 2.5 mM glucose buffer. This media was replaced with 1 ml of either 2.5 or 16 mM glucose buffer and incubated again for 60 minutes at 37 C. The insulin concentration in each solution was measured by enzyme linked immunoassay and then normalized to the DNA content of the well. We next calculated a stimulation index comparing the insulin secreted at 16 mM Glucose to the insulin secreted at 2.5 mM Glucose (Fig. 8).

There was a statistically significant increase (p < 0.05 paired t test) in the insulin secretion rate when 2.5 mM (basal) and 16 mM glucose (stimulated) were compared. The stimulation indices for all conditions were about 1.3 ± 0.2. The insulin secretion rates were less on day 7 (following 48 hr incubation in culture media supplemented with 4 mM glucose) relative to day 7.

**Figure 9:**
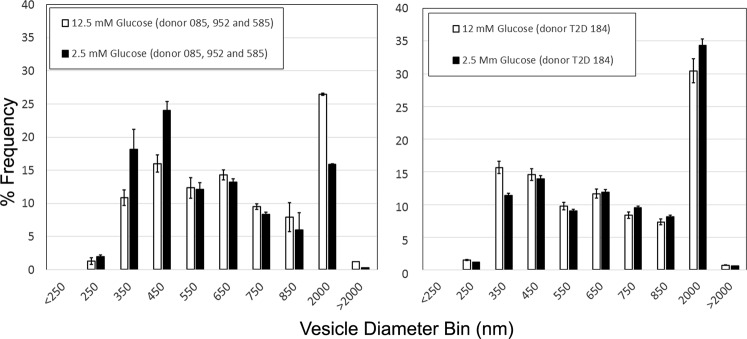
Frequency distribution of vesicle diameters in human β-cells cultures from three donors varies according to culture glucose concentrations. The OpenCFU outputs of vesicle counts and diameters were binned (in intervals of 100 nm starting at 250 nm and ending at 2500 nm) and the frequency distribution histograms prepared. Automated vesicle counts ranged from 300–800 per cell, total measurements ranged from about 9k to 15 k vesicles per culture. Experiments performed on three different healthy donors and one donor diagnosed with T2D (085, 952, 585 and 184(T2D)).

To demonstrate the utility of identifying and quantifying VMAT2 positive, zinc positive vesicles in living β-cell cultures, we prepared β-cell cultures from two nondiabetic control donors and one donor diagnosed with type 2 diabetes mellitus (T2D). Cell cultures were either maintained in high glucose (12 mM) media or incubated in low glucose (2.5 mm) media for one-hour prior to staining with (+) DDTBZ and FluoZin-3. Next, the cell cultures were imaged, the (+) DDTBZ and FluoZin-3 channels combined and the diameters of (+) DDTBZ and FluoZin-3 double positive β-cell vesicles quantified by the OpenCFU software (see Supplemental Fig. S8). During the one hour 2.5 mM low glucose incubation period, we estimated that in control β-cells, the mean vesicle diameter is significantly reduced by about 100 nm (two-tailed t-test, p < 0.0001), relative to the diameters measured under the 12 mM glucose culture conditions (mean vesicular diameter = 734 nm) (Fig. 9, Left Panel). Vesicle diameters were not reduced by 2.5 mM glucose culture in β-cells obtained from the donor diagnosed with T2D, rather a significant increase in mean vesicular diameter, from 688 nm to 724 nm (two-tailed t-test,p < 0.0001) was observed (Fig. 9, Right Panel).

## Discussion

We report on the synthesis, physiochemical and biological characterisation of a fluorescent ligand for VMAT2 and demonstrate its utility in an application relevant to β-cell biology. (+) DDTBZ differs from previous optical tracers used in CNS neurotransmission studies. These tracers, namely fluorescent false neurotransmitters^10,11^ and dansyl dopamine^32^, are nonselective VMAT1 and VMAT2 substrates. Future studies should include a side by side comparison of these optical tracers in both the β-cell and neuronal cell cultures to understand their similarities and differences.

The application selected to showcase the utility of (+) DDTBZ was a morphometric study of insulin secretory vesicles in cultured β-cells. Our principle findings are summarised as follows; (1) (+) DDTBZ and dopamine staining reveal a morphologically heterogeneous population, in terms of cell size, shape, vesicle number, size and contents, (2) VMAT2 and Zn^+2^ colocalise in wide range of β-cell acidic vesicle sizes, (3)The larger β-cell vesicles are enriched for proinsulin, whereas the smaller vesicles predominantly contain the processed mature insulin and both of these vesicles types, immature and mature, colocalise with VMAT2, 4) In β-cell cultures obtained from nondiabetic donors, incubation at non-stimulatory glucose concentrations promotes a shift in vesicle diameter towards the more mature insulin vesicles (around 300 nm) at the expense of the larger immature insulin secretory vesicle population. Low glucose induced vesicle maturation was not observed in the β-cell cultures obtained from the donor with T2D.

Transmission and scanning electron microscopy of β-cells *in situ* reveals about 9–13,000 secretory vesicles per β-cell with mature dense core vesicles having an average diameter of 300 nm^33–35^. In these studies, immature vesicles, staining less avidly with heavy metal ions (a.k.a. grey granules) have been reported but never studied morphometrically as detailed in this report. In our *in vitro* system, we found only 20–40 percent of the vesicles within the β-cell population in the diameter range of mature granules (≤390 nm). A second difference between our results and previous findings is the number of vesicles. We counted (either manually or by computer assisted image analysis) no more than 1,000 vesicles per cell staining for (+) DDTBZ and FluorZin-3. The simplest explanation for this difference is that our lower vesicles counts are due to the smaller cell volumes encountered in this “flattened β-cell” culture model. *In situ* β-cells are estimated to have a 10 um diameter^36^ corresponding to a spherical volume of 525 µm^3^. The volumes of β-cells characterised in this system are better estimated by prolate ellipsoids with x, y and z axes of approximately 36, 12 and 0.25 µm, respectively, corresponding to a volume approximately one-tenth that reported for *in situ* β-cells. Hence, our lower vesicles counts are concordant with previous reports when vesicle counts are normalised to cell volume. The flattened and dissociated β-cells studied in this system displayed GSIS stimulation indices (S.I.) that where low (S.I. ≈ 1.3) relative to freshly isolated islets, yet equivalent to reports of islets that have been cultured for five days^37^.

*In vitro* human beta single cell imaging with (+) DDTBZ reveals significant heterogeneity. Single β-cell heterogeneity has been previously documented for variety of outcome measures using different experimental systems including flow cytometry (reviewed by Nasteka and Hodson^38^). Most recently VMAT2 has been shown to be a marker of immature β-cells in cultures of induced pluripotent stem cells and its specific inhibitor, DTBZ, been shown to be an important regulator of β-cell maturation and β-cell mass^39–41^. The ability to probe heterogeneous populations and identify living immature β-cells by (+) DDTBZ staining may be a useful application of this probe.

## Materials and Methods

### Chemicals

Enantiomerically pure (+)-9-O-Desmethyl-α-Dihydrotetrabenazine was obtained from BOC Sciences, Long Island, NY and represented a convenient starting material for synthesis. All organic synthetic reagents were obtained from Sigma Millipore (St. Louis, MO). Racemic Dihydrotetrabenazine (DTBZ) was obtained Santa Cruz Biotechnology (Santa Cruz, CA, sc-220288) All other reagents were of the highest commercially available quality.

### Synthesis of (+) DDTBZ

(+)-9-O-Desmethyl-α-Dihydrotetrabenazine (20 mg, 0.06 mmol) was dissolved in 2.5 mL dichloromethane and a catalytic amount of 4-Dimethylaminopyridine (DMAP) was added. The reaction was cooled to 0 °C and slowly, drop-by-drop, dansyl chloride (17.5 mg, 0.06 mmol) dissolved in 2.5 mL dichloromethane was added. Reaction was stirred for 2 h at 0 °C and for 24 h at room temperature. Reaction mixture was concentrated under vacuum and purified using flash chromatography in 1% methanol/dichloromethane solvent system. The product 2-hydroxy-3-isobutyl-9-methoxy-1,3,4,6,7,11b-hexahydro-2H-pyrido[2,1-a]isoquinolin-8-yl 5-(dimethylamino)naphthalene-1-sulfonate ((+) DDTBZ) was obtained as a yellow-green oil.

### Purification and analysis of (+) DDTBZ probe

Column flash chromatography was carried out using E. Merck silica gel 60 F (230–400 mesh) (Sigma Millipore). The proton NMR spectra of (+) DDTBZ, recorded at room temperature on a Varian-NMR spectrometer, was difficult to interpret, presumably due to the presence of rotamers. A tentative assignment, based on the previously published spectra of tetrabenazine^42^, is a follows: ^1^H NMR (CDCl_3_, 400 MHz) δ: 0.8 (6 H,m,R-C**H**_3_), 1.3–1.8 (8 H, m, R-C**H**_2_-R), 1.9 (1 H,s, R_3_-C**H**), 2.8 (6 H,m, RN(C**H**_3_)_2_), 3.2 (1 H, d, -O**H**), 3.8 (3 H,d,-OC**H**_3_), 6.6–8.6 (8 H, m, Ar**H**) (referenced to internal Me4Si at δ_Η_ O ppm). The high performance liquid chromatography (HPLC) system: a Shimadzu LC-6AD pump equipped with an SPD-M10A PDA detector was used and a single Hamilton Co. (Reno Nevada) manual injector. Semi-preparative chromatography was performed using SymmetryPrepTM C-18 C18 7 μm, 19 × 150 mm column, (Waters). Mobile phase details: Buffer A was composed of distilled water, and buffer B 0.25% acetic acid in 90% acetonitrile. Purity (>96%) was confirmed by analytical LC/MS recorded with a Shimadzu system. Elution started with water (95%, +0.1% formic acid) and acetonitrile (5%, +0.1% formic acid) and ended with acetonitrile (95%, 0.1% formic acid) and water (5%, 0.1% formic acid) and used a linear gradient at a flow rate of 0.2 mL/min.

ESI-MS spectra were obtained using a mass spectrometer Agilent ifunnel Q-TOF 6550 LC–MS with source Dual Agilent Jet Stream ESI (Dual AJS-ESI). The solution of the samples was directly injected into the ESI source using an autosampler FIA at flow rate of 1 µL min^−1^ at 25 °C. The solutions were eluted in a gradient of 20% water (0.1% formic acid) and 80% methanol (0.1% formic acid) for ESI(+) spectra. Mass spectra of the compounds by ESI-MS were acquired using the following operating conditions: capillary voltage ±3 kV, drying gas flow 10 L min^−1^, drying gas at 250 °C, nebulizer gas at 50 psi. The system was operated for acquisitions in the mass range of 50–1500 m/z.

### Characterisation of UV-Visible emission and absorption profiles of (+) DDTBZ

Absorption spectra in the 250–750 nm range were recorded on a Biochrom WPA 80-3003-75 Model Biowave II UV/Visible Life Science Spectrophotometer. Emission spectra were recorded on a Thermoscientific Lumina Fluorescence spectrometer, using a 150 W Xenon Lamp and the excitation source set to the absorbance maximum of the compound in the 300–700 nm range. The spectral bandwidth was 5 nm, with a 2 second integration time. Data were acquired with Luminous software.

### Cell Culture

The cell lines HEK 293, PANC1, βTC-6 and 5637 were obtained from the ATCC (Manassas, VA) and cultured according to the supplier’s recommendations. HEK-DAT/mCherry-VMAT2 transfectant cells were obtained as a kind gift from Dr. G. Miller (Emory University, Atlanta, GA) and have been fully described elsewhere^43^. These cells were cultured at 37 °C and 5% CO_2_ in DMEM based selection media, supplemented with 10% FBS, 1% GlutaMax solution and 100 µg/ml Zeocin (In vivoGen, San Diego, CA and 500 µg/ml G418 (Millipore Sigma, St. Louis, MO).

Columbia University Medical Center Institutional Review Board approved human islets, isolated from cadaveric nondiabetic and diabetic donors, were obtained from the Integrated Islet Distribution Program (IIDP) (City of Hope National Medical Center). The average purity and viability of the islets obtained from control donors (n = 9) was 89 ± 2% and 95 ± 1% (SEM), respectively. The average age and body mass index of the donors was 47 ± 3 years and 32 ± 3 kg/m^2^ (SEM), respectively. Upon receipt, the isolated human islets were cultured in supplemented CMRL-1066 media for no longer than 1 day, before being subcultured as previously described^15,44^. Briefly, human islets were washed and dissociated in 0.05% Trypsin/EDTA solution. The dissociated islets were filtered through a sterile 40 µm nylon filter and collected and washed in minimum essential medium (MEM) with GlutaMAX (Gibco), 12 mM glucose, 5% FBS, 1 mM sodium, pyruvate, 10 mM HEPES, 100 U/ml Penicillin and 100 U/ml Streptomycin and 1x B-27 Supplement (Gibco, Gaithersburg, MD). Cells were seeded at 10,000–20,000 cells/cm^2^ on Collagen IV (Millipore Sigma) coated coverslip chambers (Cellvis, Mountain View, CA) in the same medium and allowed to reach full adherence (4–5 days) before live cell imaging or immunohistochemistry. Newborn porcine islets were prepared as previously described^45^ and processed for two dimensional culture as described above, with the exception that Hams F10 media was used for culture.

In some experiments, 293 HEK or HEK-DAT/mCherry-VMAT2 transfectant cells were seeded into Collagen IV coated coverslip chambers or sterile black 96-Well Glass Bottom Microplates (Greiner Bio-One SensoPlate) previously coated with poly-L-Lysine and cultured in their corresponding media to approximately 85% confluence or chambered coverslips. Cell were incubated in “imaging media” consisting of phenol-red free RPMI 1640. media (Caisson Labs, Smithfield, UT) supplemented with 95 mg/100 ml defatted bovine serum albumin (BSA), 1 ml/100 ml Pen/Strep 100 x solution (Corning), 84 mg/100 mls Sodium Bicarbonate, 5 mM HEPES, and 12, 5 or 2.5 mM Glucose) supplemented with the fluorescent probe and/or (+) DTBZ at the indicated concentrations. Cultures were incubated in the dark for 45 minutes at room temperature, washed twice with imaging media without probes, and then incubated in probe-free imaging media for a further 30 minutes at 37 °C in a humidified 5% CO_2_ atmosphere.

### Fluorescent ligand binding experiments

Semi confluent 293 HEK or HEK-DAT mCherry-VMAT2 transfectant cell cultures, seeded into 96 well plates, were washed once in room temperature imaging media. Supernatants were then replaced with imaging media supplemented with (+) DDTBZ and (+) DTBZ at the indicated concentration and further incubated as described above. Cell associated fluorescence measurements were performed on a Biotek Synergy 2 instrument, using the following parameters: (+) DDTBZ, excitation at 320 nM with a 40 nm band pass filter, emission at 500 nm with 20 nm bandpass filter, mCherry fluorescent protein, excitation at 530 nM with a 25 nm band pass filter, emission at 590 nm with 35 nm bandpass filter. The cell bound fluorescence signal from DDBZ was normalised to the intensity of VMAT2-mCherry 590 nm fluorescence signal which served as a surrogate measure of cell number per well.

### Microscopy and Live Cell Imaging

A Zeiss Axiovert 135 inverted epifluorescence microscope fitted with two independently controlled collimated light sources, 5.1 W Solis 385 nm LED or a 4.2 W Solis 400–700 nm LEDs (Thorlabs Inc., Newton, NJ) and Zeiss a 50x LD EC Epiplan-Neofluar (0.55 NA), 63 x A Plan (0.8) and 100x Plan Neofluar (1.3 NA) objectives were used for cell imaging. Specific excitation and emission wavelengths where obtained with the following filter sets; A) Excitation 330WB80, Dichroic 400DCLP, Emission 495BP30 (used for DAPI and (+) DDTBZ), B) Excitation 470QM40, Dichroic 505 DRLP, Emission 535QM50 (used for FluoZin-3, AM or NeuroSensor 521), C) Excitation 560QM55, Dichroic 595 DCLP, Emission 645QM75 (used for LysoTracker Red DND-99 and Nuclear Mask Deep Red) (all from Omega Optical, Brattleboro, VT). (+) DDTBZ was imaged first using the 385 nm source. The 385 nm source was then extinguished and the broadband 400–700 nm source turned on for subsequent image acquisition. In this optical system, at 100 x magnification under oil, the x-y resolution was around 220 nm (given by the Abbe limit) and the depth of field of approximately 250 nm (given by the Shillaber equation). Under these culture conditions, most β-cells could be captured in their entirety using a single focal plane.

Images were projected onto a Lumenera Infinity 2 monochrome 2 megapixel camera (Ottawa, Ontario, CA) and acquired with Lumenera Infinity Capture or IMAGEJ software. Image data was exported as single 1392 × 1040 pixel, 16 bit grey scale TIFF files and further processed using IMAGEJ (including colocalisation scoring)^46^, OpenCFU^47^ and/or Adobe Photoshop CS3. The size of objects within the images captured was obtained via calibration of ImageJ software with a stage micrometer (0.1 mm in 0.002 mm divisions)(Ted Pella, Inc, Redding, CA).

Cover glass chambered β-cell cultures were removed from the incubator and the culture media aspirated, washed twice with imaging media. Cultures were then incubated in imaging media (5 mM glucose) containing 30 uM (+) DDTBZ for 45 min at room temperature, washed twice in imaging media and then incubated in imaging media for a further 30 minutes at 37 °C in a humidified 5% CO_2_ atmosphere. In the indicated experiments, prior to staining, β-cell cultures were preincubated in imaging media containing 12 mM glucose or 2.5 mM Glucose for 1 hr at 37 °C, with 5% CO_2_. Coverslip chamber cultures were then moved to the microscope environmental chamber with a temperature regulated heated stage and an objective warmer ((CSH-1 and OW, Warner Instruments, Hamden, CT). The cultures were maintained at 37 °C in 5% CO_2_ in this chamber. Cells were imaged at the indicated wavelengths. In some experiments, in addition to (+) DDTBZ, cultures were also stained with 50 nM LysoTracker® Red DND-99 (Molecular Probes/Thermo Fisher Scientific, Waltham, MA), 2 µM FluoZin™-3, AM, 5 µM BODIPY™ FL C5-Ceramide complexed to BSA (both from Invitrogen/Thermo Fisher Scientific, Waltham, MA), or 100 nM NeuroSensor 521 (Millipore Sigma). Throughout the 1–2 hour imaging session, we observed no signs of toxicity such as cell rounding and detachment. Identical cultures treated with reserpine (10 µM), a fluorescent but irreversible VMAT2 inhibitor, showed cell rounding and detachment within 30 minutes of treatment.

### Immunohistochemistry

Monolayers of pancreatic islet cells were washed in phosphate buffered saline (PBS) and fixed with 4% EM-grade paraformaldehyde in PFA in PBS (Electron Microscopy Sciences) at room temperature for 15 minutes. Samples were permeabilised in PBS + 0.3% Triton X-100 and blocked with PBS + 0.2% Triton X-100 with 10% goat or rabbit serum for 60 minutes at room temperature. Primary antibodies (listed below) were incubated overnight at 4 °C. Alexa Fluor conjugated secondary antibodies (Invitrogen) were incubated in 0.3% Triton X-100 in PBS plus BSA for 60 minutes at room temperature. Coverslips were mounted with Vectasheild HardSet Antifade Mounting Medium with DAPI (Vector labs, Burlingame, CA). The following primary antibodies and dilutions were used for immunofluorescent staining: anti-Insulin rabbit monoclonal antibody, 1:1000 (Boster, #M00067-1), Human/Mouse Proinsulin Alexa Fluor® 488-conjugated mouse monoclonal antibody, 1:200 (IC13361G) and Human VMAT2 Alexa Fluor® 594-conjugated mouse monoclonal antibody, 1:200 (FAB8327T) (both from R and D systems, Minneapolis, MN). A goat anti-rabbit IgG (H + L) cross-adsorbed secondary antibody, Alexa Fluor 430 conjugate, 1:1000 (Invitrogen, #A-11064) was used to detect the rabbit primary antibody above.

### Glucose Stimulated Insulin Secretion Assays

The human beta cell cultures (prepared from two donors 324 and 228/8 wells each) were studied on day 5 (in 12 mM Glucose) and day 7 (last two days of incubation in 4 mM Glucose). The cultures were washed 4 x with Krebs Buffer Solution (HEPES 25 mM, NaCl 115 mM, NaHCO_3_ 24 mM, KCl 5 mM, MgCl_2_ 1 mM, CaCl_2_, 2.5 mM, BSA 0.1% w/v with 2.5 mM Glucose. The culture chambers were next filled with 1 ml of the warmed Krebs 2.5 mM glucose solution and the cultures were incubated (5% CO_2_) for 1 hr at 37 C degrees (similar to the protocol described for the vesicle morphometry). Next, the Krebs buffer solution was removed and replaced 1 ml Krebs buffer solution containing either 2.5 mM glucose or 16 mM Glucose and incubated again at 37 C. Sixty minutes later the Krebs buffer solution was harvested into labelled tubes. Residual Krebs buffer was aspirated and replaced with 250 ul of warm 5% SDS. The chambers were incubated for 30 min at 37 C and then the SDS solution was harvested into labelled tubes. Insulin assays were performed using an ALPCO human insulin ELISA following the manufactures’ recommendations. The DNA content of the well was measured using the Promega Quantitiec DNA assay following the manufactures recommendations. Aborbance and fluorescence measurements were made using the Synergy 2 multiplate reader (Biotek). The mean normalized insulin concentrations in the harvested Krebs buffer were calculated from replicate chambers and triplicate determinations of insulin concentration in the sample. Each chambers mean insulin concentration was normalized to the DNA content of the well.

### Data analysis

Descriptive statistics, means, modes, standard deviations, standard errors as well as frequency distributions were calculated by Excel. Statistical significance was accepted at p values less or equal to 0.01. For vesicle morphometry, we used the open source program OpenCFU on a windows platform^47^ which outputs its measurements in an Excel compatible format. Images from 20–40 cells from each culture condition and donor were acquired with the 100 x objective and resulted in diameter measurements from about 10^4^ vesicles from each human islet culture condition.

## Supplementary information

SUPPLEMENTARY INFO

## Data Availability

The datasets analysed during the current study are available from the corresponding author on reasonable request. Limited amounts of (+) DDTBZ are available from the corresponding author on request.
