# Supplementary material for: A novel optical tracer for VMAT2 applied to live cell measurements of vesicle maturation in cultured human β-cells
Source: Sci Rep. 2019 Apr 1;9:5403. doi: 10.1038/s41598-019-41891-x (PMC6443945; doi:10.1038/s41598-019-41891-x)
Supplement: Supplementary file 1 — SUPPLEMENTARY INFO [file 41598_2019_41891_MOESM1_ESM.pdf]

Supplemental Figures for "A novel optical tracer for VMAT2 applied to live cell measurements of vesicle maturation in cultured human β-cells.

Authors: Pecic, Stevan, Milosavic, Nenad, Rayat, Gina, Maffei, Antonella and Harris, Paul E.

Supplemental Figure S1.

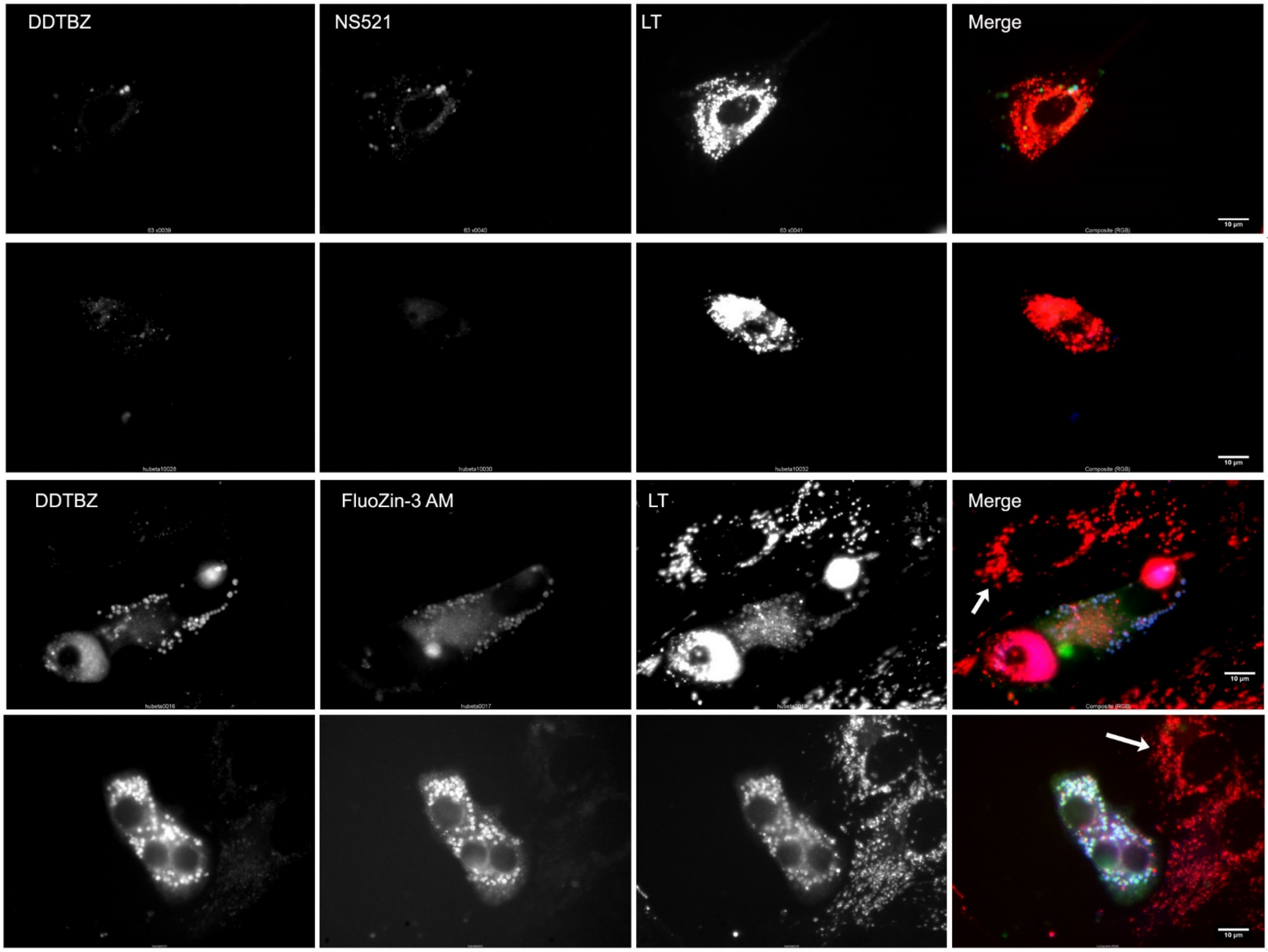

Supplemental Figure S1. A subset of cells in islet cultures do not bind stain well for (+) DDTBZ, NeuroSensor 521 or FluoZin-3 AM yet stain for LysoTracker® Red DND-99. Cell cultures were stained with the three probes and imaged in their corresponding channels. Images obtained from two cell cultures from islet donors 025 and 141. The merged pseudocolour image (far right panels) represents the three grey scale images, (+) DDTBZ (blue channel), NeuroSensor 521 or FluorZin-3 AM (green channel) and Lystotracker Red DND-99 (red channel) translated to an RGB image. White arrows identify (+) DDTBZ and FluorZin-3 AM weak/negative cells. Scale bar represents 10 µm.

Supplemental Figure S2

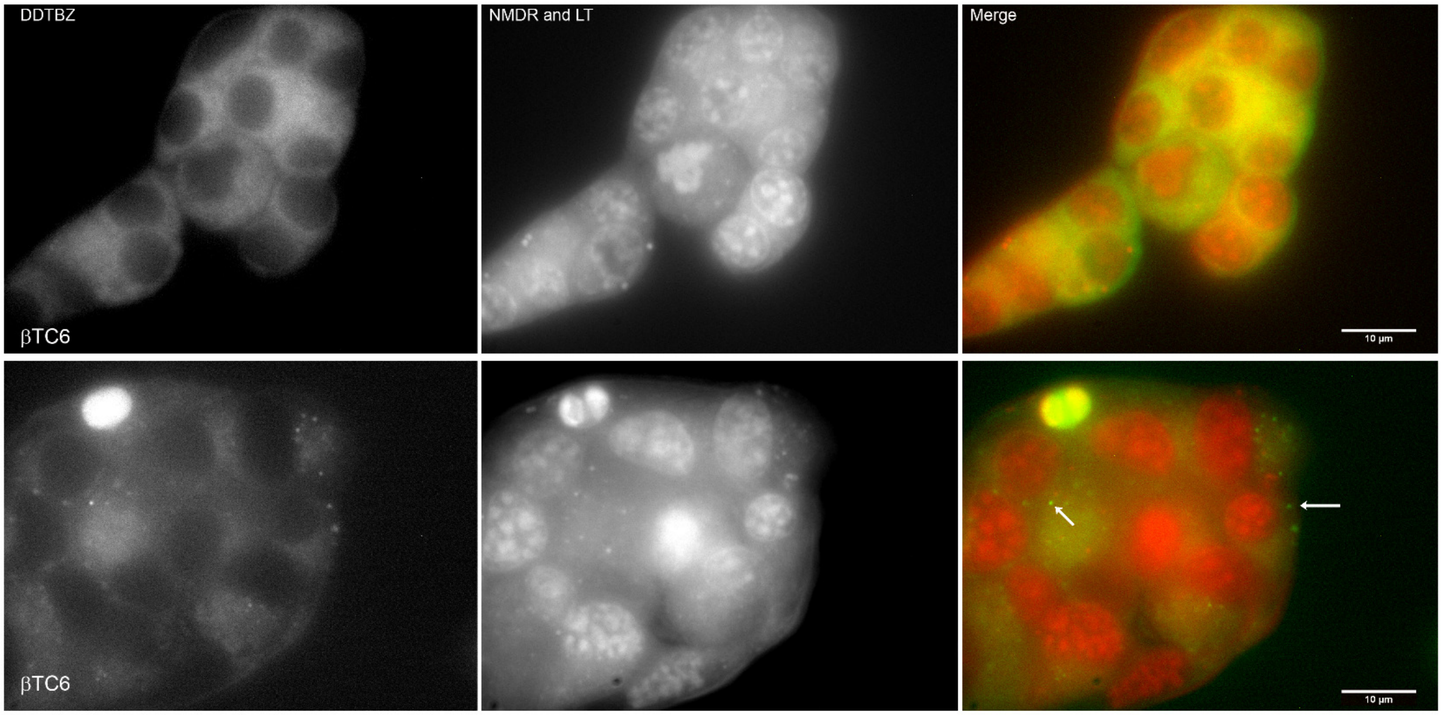

Figure S2. The βTC-6 murine cell line is heterogeneous with respect to vesicular binding of (+) DDTBZ. βTC-6 were stained with (+) DDTBZ, Nuclear Mask Deep Red and Lysotracker. The (+) DDTBZ signal was acquired with a 495BP30 filter and is shown in green in the pseudocolour merged images at right. Nuclear Mask Deep Red and LysoTracker Red DND-99 signals were imaged together in a 610 to 700 nm window and are shown in red in the pseudocolour merged images at right. White arrows indicate vesicles staining with (+) DDTBZ. Scale bar represents 10 microns

Supplemental Figure S3

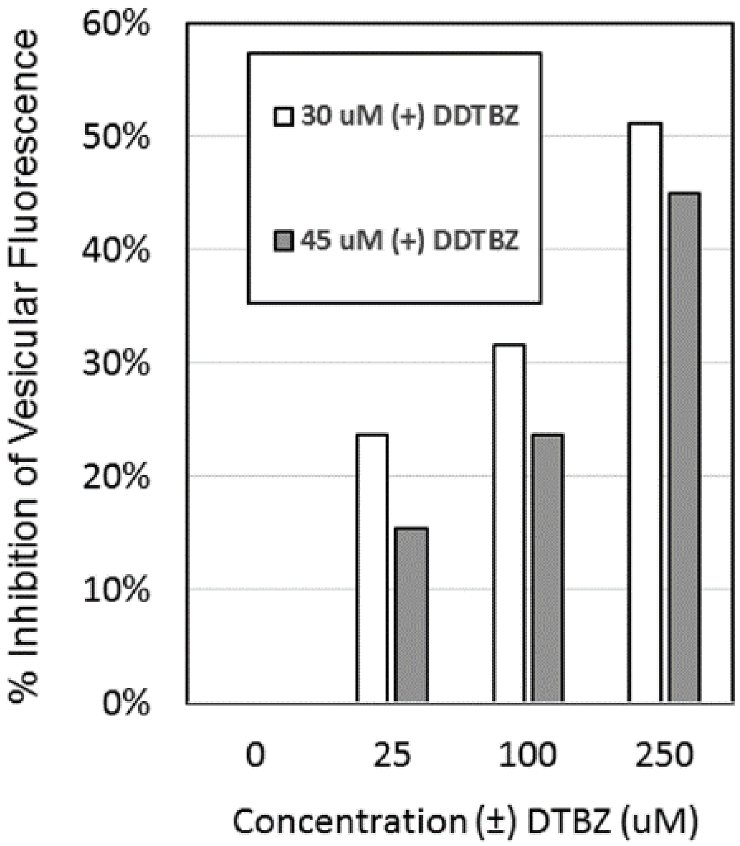

Supplemental Figure S3. Inhibition of (+)DDTBZ vesicular fluorescence by (±) DTBZ. Human islet β cell cultures were stained with (+) DDTBZ and the indicated concentration of racemic (±) DTBZ for 45 minutes at room temperature and then washed. Cultured cells were then imaged at 100 x . Twenty to 40 cells (corresponding to > 1000 vesicles) per condition were imaged with the following filter set Excitation 330WB80, Dichroic 400DCLP, Emission 495BP30. Cellular vesicles were identified by OpenCFU software and vesicular signal intensity was calculated for each vesicle as the quotient of the mean pixel intensity and vesicle pixel area. Mean vesicular signal intensities for each condition and the percent inhibition relative to cultures treated only with (+) DDTBZ were calculated. All reported values were significantly different ($p<0.05$) from one another. Results obtained from a single islet donor (567).

Supplemental Figure S4

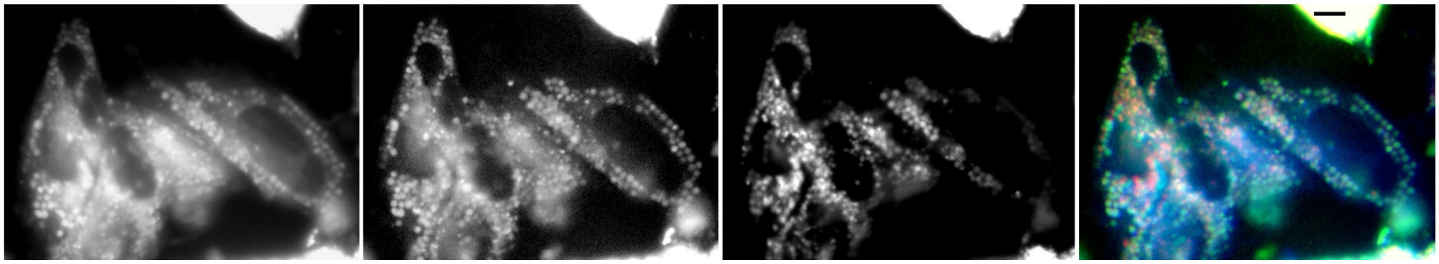

Supplemental Figure S4. (+) DDTBZ, NeuroSensor 521 and LysoTracker® Red DND-99 staining of live porcine β-cell cultures identifies VMAT2 positive, dopamine positive, acidic vesicles in a population of dispersed islet cells. Cultures were stained (Panels left to right) with (+) DDTBZ, NeuroSensor 521 and LysoTracker® Red DND-99 and imaged in the blue, green and red channels as described in Figure 6. Scale bar in the image at the far right represents 10 µm.

Supplemental Figure S5

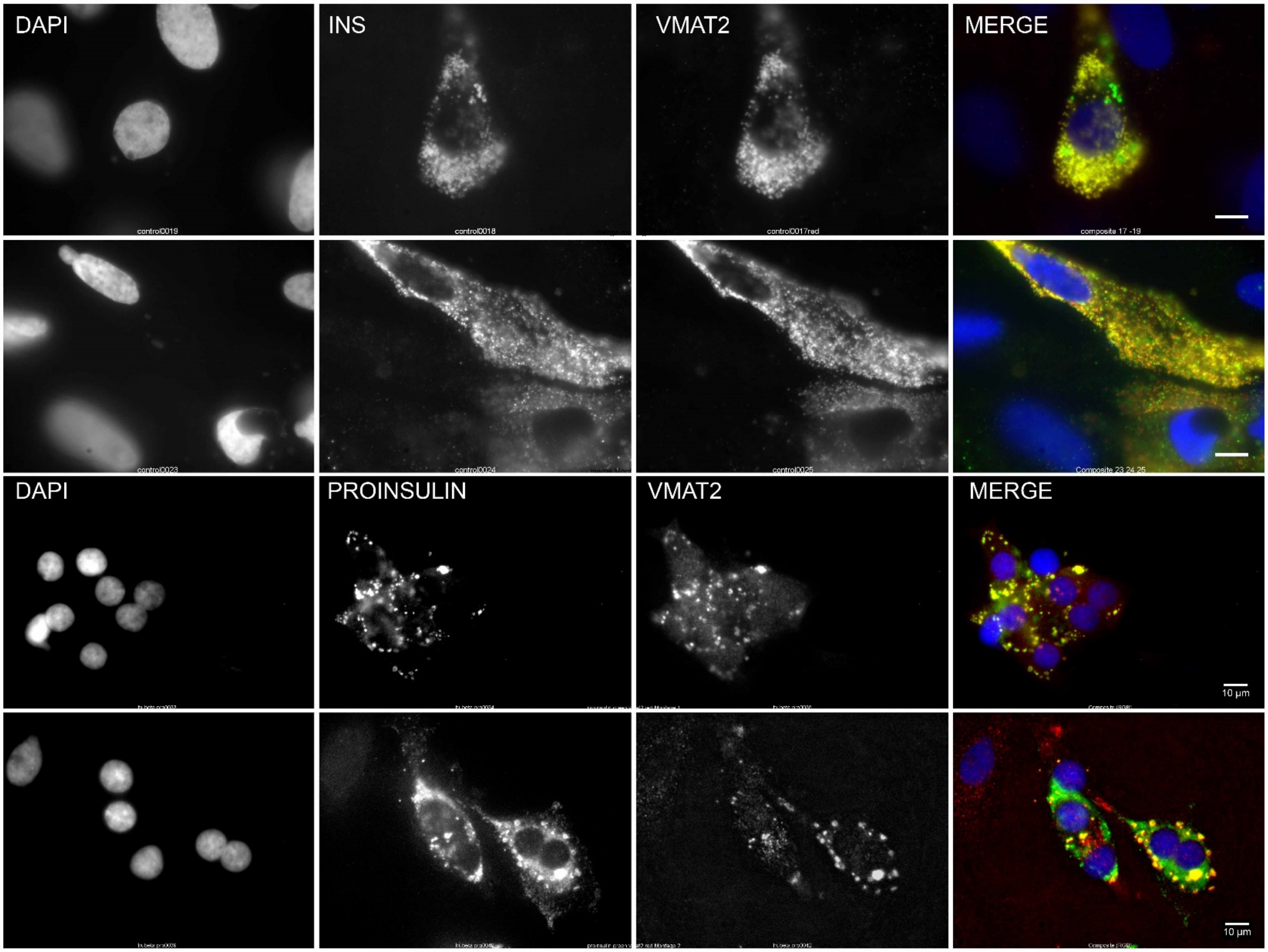

Supplemental Figure S5. Insulin and proinsulin colocalise with VMAT2 in the vesicles of cultured human β-cells. Cultures of human islet cells (donor 212) were fixed for immunohistochemistry with stained with anti-insulin, anti-proinsulin and anti VMAT2 antibodies. Nuclei were counterstained with DAPI and imaged. The far right panels are the merged pseudocolour images from the single focal plane obtained from photographs prepared in each channel (DAPI-blue, insulin, proinsulin-green, VMAT2-red). Scale bar represents 10 μm

Supplemental Figure S6

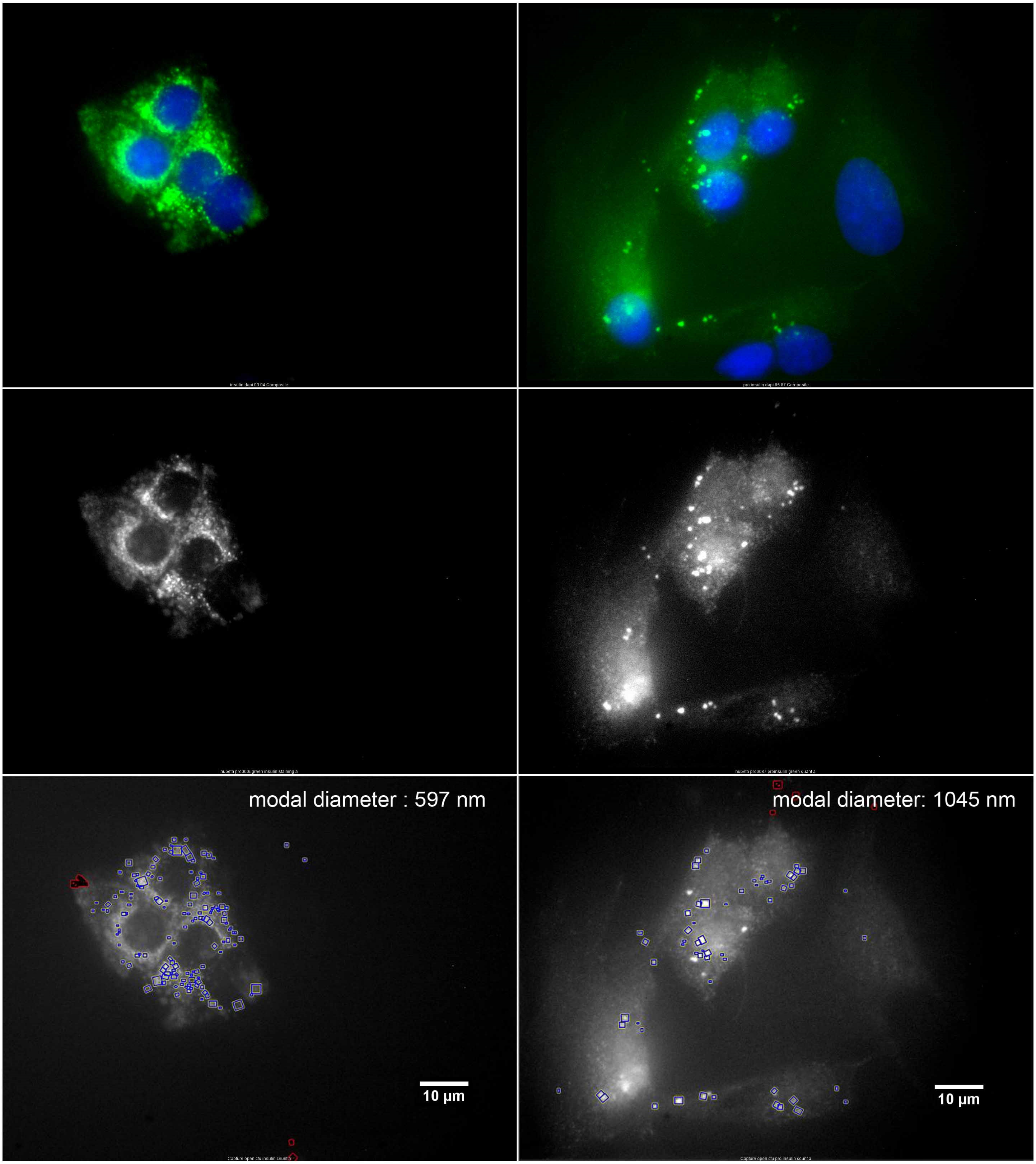

Supplemental FigureS6. β-cell vesicles containing proinsulin are larger in diameter than vesicles containing insulin. Cultures of human β-cells (from donor 212) were fixed for

immunohistochemistry with stained with anti-insulin (Left Panels) or anti-proinsulin (Right Panels) and nuclei counterstained with DAPI and imaged (Top Panels). The grey scale insulin or proinsulin images (Middle Panels) were processed by OpenCFU to obtain measurements of the particle diameters identified by the program and bounded by blue boxes (Bottom Panels). Scale bar represents 10 µm.

Supplemental FigureS7.

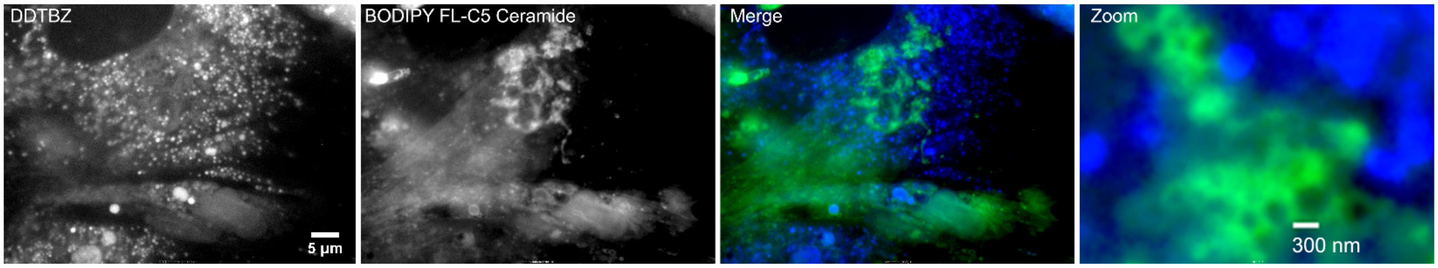

Supplemental Figure S7. (+) DDTBZ and BODIPY FL C5-Ceramide staining identify nascent and more mature human β-cell vesicles. Cytoplasmic areas relative devoid of (+) DDTBZ avid vesicles stain well with BODIPY FL C5-Ceramide to reveal the trans-Golgi network. (+) DDTBZ negative vesicles still associated with the network are visible in the zoomed panel of the pseudocolour merged image ((+) DDTBZ-blue, BODIPY FL C5-Ceramide-green). Images are representative from β-cell cultures obtained from 2 donors (567 and 256).

Supplemental Figure S8.

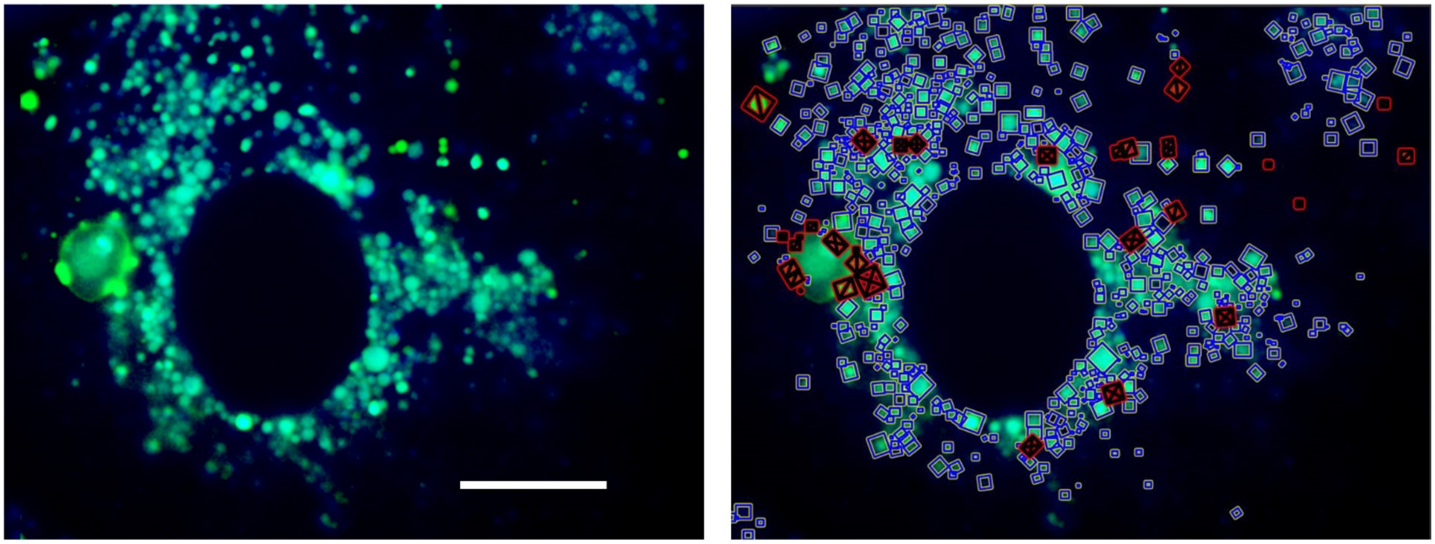

Supplemental Figure S8. **OpenCFU vesicle counting in images of live human β-cell cultures stained with (+) DDTBZ and FluoZin™-3 AM zinc probe**. Left panel. Merged imaged of (+) DDTBZ channel (blue) and FluoZin™-3 AM zinc probe channel (green). Right Panel. Vesicles identified and counted by Open CFU program used in the vesicle morphometry studies in Figure 8. Five hundred four vesicles were identified by OpenCFU versus a manual count of 463. Scale bar represents 10 µm.
